# Insights into the Anaerobic Hydrolysis Process for Extracting Embedded EPS and Metals from Activated Sludge

**DOI:** 10.3390/microorganisms9122523

**Published:** 2021-12-06

**Authors:** Barbara Tonanzi, Agata Gallipoli, Andrea Gianico, Maria Cristina Annesini, Camilla Maria Braguglia

**Affiliations:** 1 Water Research Institute, National Research Council of Italy, IRSA-CNR, Area della Ricerca RM1, Via Salaria km 29.300, Monterotondo, 00015 Roma, Italy; barbara.tonanzi@irsa.cnr.it (B.T.); andrea.gianico@irsa.cnr.it (A.G.); camilla.braguglia@irsa.cnr.it (C.M.B.); 2 Dipartimento di Ingegneria Chimica, Università degli Studi di Roma “La Sapienza”, 00184 Rome, Italy; mariacristina.annesini@uniroma1.it

**Keywords:** anaerobic hydrolysis, extracellular polymeric substances, waste activated sludge, toxic metals, high throughput 16S rRNA gene sequencing, microbial communities

## Abstract

The amount of sewage sludge generated from wastewater treatment plants globally is unavoidably increasing. In recent years, significant attention has been paid to the biorefinery concept based on the conversion of waste streams to high-value products, material, and energy by microorganisms. However, one of the most significant challenges in the field is the possibility of controlling the microorganisms’ pathways in the anaerobic environment. This study investigated two different anaerobic fermentation tests carried out with real waste activated sludge at high organic loading rate (10 g COD L^−1^d^−1^) and short hydraulic retention time (HRT) to comprehensively understand whether this configuration enhances extracellular polymeric substance (EPS) and metal solubilisation. The quantity of EPS recovered increased over time, while the chemical oxygen demand to EPS ratio remained in the range 1.31–1.45. Slightly acidic conditions and sludge floc disintegration promoted EPS matrix disruption and release, combined with the solubilisation of organically bound toxic metals, such as As, Be, Cu, Ni, V, and Zn, thereby increasing the overall metal removal efficiency due to the action of hydrolytic microorganisms. *Bacteroidetes*, *Firmicutes*, and *Chloroflexi* were the most abundant phyla observed, indicating that the short HRT imposed on the systems favoured the hydrolytic and acidogenic activity of these taxa.

## 1. Introduction

Wastewater treatment plants (WWTPs) are typically considered as facilities in which contaminated water is treated to produce a clean effluent and an inevitable semi-solid by-product (sewage sludge). However, in recent years their high potential in terms of resource recovery has gained increasing attention. One of the key components for maximising resource recovery in a WWTP is the sludge produced through the primary and secondary treatment steps. After primary physical-chemical treatments, the biodegradable organic matter contained in the wastewater is removed by the secondary activated sludge process. At this stage, wastewater is treated by active biomass constituted by biological aggregates named flocs. The active biomass assimilates the organic matter, thus reducing the oxygen demand, and producing an excess biomass known as waste activated sludge (WAS). The virtuous shift to a more efficient resource recovery facility should integrate biochemical, physical, and biological conversion processes to extract value, if any, from complex waste streams as activated sludge. Floc-embedded microorganisms produce soluble microbial products and extracellular polymeric substances (EPS) due to biological or mechanical stress [1]. Soluble microbial products consist of cellular components released into solution during cell lysis, diffused across the cell membrane, or excreted for other purposes [2]. Furthermore, EPS typically remain within the sludge flocs. EPS are mixtures of macromolecular substances such as polysaccharides, proteins, humic and fulvic acids, lipids, and nucleic acids, which have been found to occur in the intracellular space of microbial aggregates [3,4], due to active secretion, shedding of cell surface material, cell lysis, and adsorption from the environment [2,5]. These biopolymers support several important cellular functions, such as binding of metal ions or organic compounds, water retention, and microbial adhesion to a surface [5].

Due to the nature of their association with cells and flocs, and considering the extraction techniques used to separate them, EPS can be broadly classified as soluble (“slime” polymers) and bound fractions. The bound EPS are closely attached to microbial cells or flocs, whereas the soluble EPS are able to move freely among sludge flocs being dissolved in the surrounding liquor [6,7]. These soluble EPSs are therefore identical to soluble microbial products (SMP), which are soluble cellular components that are released and dissolved into solution [2]. Both fractions are generally separated by centrifugation: the supernatant contains the soluble EPS whereas the bound EPS remain attached to the solid particles.

The EPSs in activated sludge, forming a three-dimensional matrix of aggregates as biofilms or flocs, account for 10–40% of the sludge dry weight. Moreover, 75–89% of the extracellular organic carbon can be attributed to proteins and saccharides. Extracellular carbohydrates, proteins, and nucleic acids all have binding capacities to form the complexes with heavy metals [8]. In addition, Comte et al. [9] suggested that soluble EPS had a more significant adsorptive capability for heavy metals with respect to bound EPS. EPS have received increasing attention for a number of wastewater treatment applications, such as particle flocculation, heavy metal adsorption, toxic organic chemical removal, and dye decolourisation [10]. Therefore, the extraction of EPS from sludge presents several application perspectives, including industrial ones.

Anaerobic digestion (AD) starts by breaking down the sludge structure through hydrolysis, increasing the sludge surface area and the soluble organics content [11]. Novak et al. [12] showed that the amount of proteins and carbohydrates released in the disintegration of anaerobic sludge is strongly dependent on the hydraulic retention time (HRT) in a range between 1 and 9 days. At the same time, HRT can also affect the transformation of EPS into short chain fatty acids, which have commercial applications even if they are not in their pure forms [13]. Therefore, the release of embedded EPS from biosolids through appropriate pre-treatments and/or biological hydrolysis can be very effective [14].

The anaerobic hydrolysis stage of biogenic residues poses a promising platform for the wide-spread development of biorefinery systems. More studies and investments are needed to reduce initial costs and encourage their implementation at an industrial scale for a sustainable bio-economy [15,16].

For these reasons, the objectives of this study were focused on understanding the sludge anaerobic degradation mechanisms of the hydrolysis step carried out at constant high organic loading rate (OLR) and short HRT (3 and 4 days), by investigating the release of EPS and metals, the fate of proteins and polysaccharides, and the process performances in terms of methane production and organics removal. To further understand the underlying mechanisms in such systems, the microbial community was investigated.

## 2. Materials and Methods

### 2.1. Digester Operation

Two distinct anaerobic reactors were run with the aim to study the waste activated sludge (WAS) hydrolysis. The reactors, completely mixed with a working volume of 3 L, were operated at mesophilic temperature (37°C) in semi-continuous mode, feeding sludge from Monday to Friday. Both reactors, seeded with anaerobic inoculum deriving from a full-scale digester treating sewage sludge (TS = 30.2 ± 2 g L^−1^; VS = 15 ± 3 g L^−1^; COD_tot_ = 26.3 ± 5 g L^−1^), were fed with real WAS, dynamic thickened (2000 g for 3 min) and conveniently diluted to maintain an OLR of 10 ± 3 g COD L^−1^d^−1^. Reactor I was operated at hydraulic retention time (HRT) of 4 days; Reactor II at HRT of 3 days. Table 1 shows the characteristics of the feedstocks used for R-I and R-II. Due to the duration of the tests and to different sampling periods, WAS characteristics were partly affected by seasonal effects; the sludge used as feed for R-II presented higher VS content (%TS) and lower soluble COD with respect to the one fed to R-I.

### 2.2. Analytical Procedure

APHA standard methods were used to determine total and volatile solids [17]. To analyse the soluble phase, the effluent was centrifuged (10 min at 5500 rpm) and the particulate sludge matter was removed. The resulting phase was filtrated through 0.45 μm pore size membrane filters. Total and soluble chemical oxygen demand (COD) were analysed in duplicates by means of COD Cell Test by Spectroquant Merck (EPA method 410.4). Total nitrogen was determined photometrically according to Koroleff’s method through cell tests by Spectroquant Merck. Ammonium nitrogen (NH_4_^+^-N) was determined according to APHA Standard Methods. For protein and carbohydrates determination, effluent aliquots were filtered through filters with 1.2 μm pores (GF/C Whatman). Protein content was calculated by means of the modified Lowry Kit for Protein Determination (Sigma-Aldrich P 5656, St. Louis, MO, USA). Carbohydrate determination was based on a colorimetric modified Dubois method, reported in Pagliaccia et al. [18].

Protein and carbohydrate concentrations were converted to COD equivalent by multiplying by 1.5 and 1.07, respectively [19].

### 2.3. EPS Extraction

A quantity of 500 mL effluent was centrifuged at g (~rpm) for 10 min at 4 °C. The supernatant was collected as soluble-slime EPS (sEPS) and dialysed successively against demineralised water at least eight times to remove low-molecular weight metabolites [20].

The dialysed components were freeze-dried (Alpha 1-4 LDplus, Martin Christ, Osterode am Harz, Germany) to obtain dry solids. The dried sEPS were weighed to assess the concentration in the processed effluent.

EPS recovery was based on the influent COD using the equation:

$$EPS\;recovery\;\left(\%\right)=\frac{\left(C_{eps}\times Q_{effl}\right)}{\left(COD_{inf}\times Q_{inf}\right)}$$

$$Q_{effl}=Q_{inf}$$

where *Q_inf_* and *Q_effl_* are the flow rates (L d^−1^) of the influent feed and of the effluent, respectively; *C_eps_* is the soluble EPS concentration in the effluent (g L_effl_^−1^); and *COD_inf_* is the influent COD (g L^−1^).

### 2.4. DNA Extraction and Microbial Community Analysis

Biomass was sampled from the reactors at the end of the operation and was immediately stored at −20 °C. One mL of digestate (corresponding to ~0.25 g wet weight) was used to extract DNA with the PowerSoil DNA Isolation kit (MoBio, Carlsbad, CA, USA). DNA was eluted with 100 µL sterile distilled water and the concentration and purity determined by a NanoDrop 2000 c spectrophotometer (Thermo Scientific, Waltham, MA, USA). The genomic DNA was stored at −80 °C for several days and then used for high throughput 16S rRNA gene sequencing.

16S rRNA V1-3 and V3-5 variable regions were sequenced for bacterial and archaeal community analysis, respectively. High throughput 16S rRNA gene sequencing used protocol were previously reported in Tonanzi et al. [21]. QIIME2 version 2018.2 was used to process, quality filter, and analyse the raw sequences. The demultiplexed reads were processed with the DADA2 pipeline to identify amplicon sequence variants (ASVs) [22]. Taxonomy was assigned using a pre-trained naïve-Bayes classifier based on the 16S rRNA database at a 99% similarity of SILVA132 release [23].

Total ASVs generated from each sample were used to perform the diversity analyses using the statistical software PAST 3 (version 2.17) [24]. Briefly, the Shannon index (H) measures diversity considering the number of ASVs and increases as diversity increases. Shannon diversity divided by the logarithm of the number of taxa provides the Equitability index (J). This index measures the evenness with which individuals are divided among the taxa present in the sample. Simpson’s diversity index calculates a diversity score for a community. It is based on both the number of different species in the community and the number of individuals present for each of those species. Approximate confidence intervals for all indexes were computed with a bootstrap procedure (default 9999) and a 95% confidence interval was then calculated.

## 3. Results and Discussion

### 3.1. Performance of Fermentation Reactors

Waste activated sludge, due to its biological origin, was rich in N and P, and mainly composed of particulate organics. The VS/TS ratio, which provides an indication of the organic fraction in the sludge solids, was 0.6–0.7 lower with respect to typical value ranging from 0.7 and 0.8 [25], suggesting low biodegradability due to the long sludge age of the plant.

**Figure 1:**
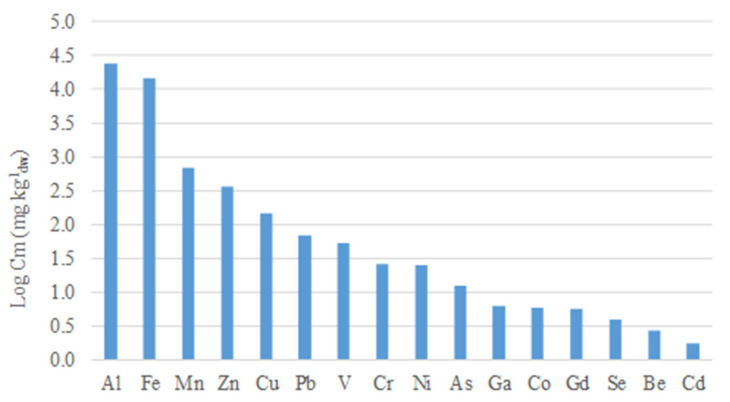
Average total concentrations (log Cm, mg kg^−1^) of heavy metals and metalloids in raw waste activated sludge used in this study.

As expected, for the six heavy metals (Cd, Cu, Hg, Ni, Pb, and Zn) having limits set out in the EU Directive for sludge utilisation in agriculture, the concentrations in the WAS were very low, and also do not pose problems in the case of inevitable metal enrichment due to solids removal of stabilisation processes (Figure 1). Additional limit values provided for the European countries are, however, very different, and almost all countries provide limit values for total chromium and, in some cases, arsenic. Italy also recently introduced (law No. 130/2018) limits for beryllium and selenium [26].

Relevant concentration of As and Se, potentially toxic metalloids found in this WAS, could be ascribed to the input coming from natural background sources typical of the territory of Central Italy, which also affected the quality of the biosolids quality to be disposed of [27].

**Figure 2:**
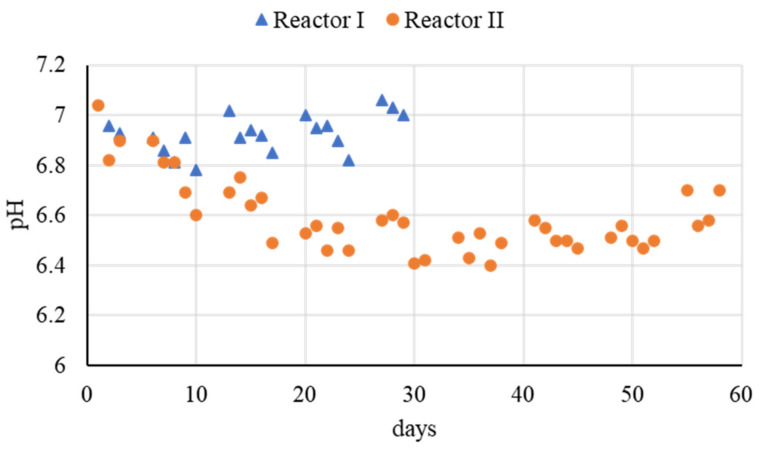
Comparison of pH value between R-I and R-II during the entire operation.

Fermentation-based anaerobic bioleaching process was carried out in order to investigate the efficiency of biological hydrolysis and acidification to extract these metals, together with the EPS, from sludge (see Section 3.2). Fermentation performance was evaluated at mesophilic temperature at an OLR of 10 ± 1 g COD L^−1^d^−1^ and HRT of 4 days (Reactor I) and 3 days (Reactor II). The OLR applied was approximately four times higher than that of a typical anaerobic digester. The pH value of R-I showed a slightly decreasing trend over the week (because of daily feeding that promoted acidification of the system) but recovered to neutrality during the weekend (Figure 2). On the contrary, by decreasing the HRT to 3 d (Reactor II), the pH continuously decreased to 6.5 during the first 3 weeks of operation (approximately 5 HRT), before remaining stable and slightly acidic until the end of the test (Figure 2).

An accumulation of propionate and acetate was observed in Reactor II, with an acidification degree of 23 ± 2%. Conversely, in Reactor I the acidification degree was lower (only 9%) due to the removal of VFAs during the process. The accumulation of acetic and propionic acids in Reactor II could be attributed to the instability of the methanogenic process due to the low HRT applied.

The performances in terms of methane production and solids reduction confirmed this trend: the specific methane production of Reactor I was significantly higher, namely 0.07 against the 0.045 LCH_4_ g^−1^VS_fed_ of Reactor II, with a VS removal of 25% (against 10% of Reactor II). These low methane yields confirmed the impact of short HRTs on methanogens [28].

### 3.2. Solubilisation of Organics and Metals from Sludge Disintegration

Table 2 summarises the main substances detected in the reactor effluents during the fermentation process. It can be seen that after the start-up period the effluents contained higher proteins, polysaccharides, phosphorus, NH_4_^+^, and VFAs than the raw WAS supernatant (see Table 2) due to sludge disintegration and hydrolysis processes occurring in anaerobic fermentation. These variations were in agreement with other authors’ observations [29,30,31].

The increase in carbohydrate and protein contents in both reactors indicated that the degradation rates of these compounds were lower than their release rates from sludge flocs, in particular for the protein fraction. Nevertheless, a significant portion of the soluble COD in R-I was removed between days 17 and 30 due to VFA’s transformation into methane, which increased significantly.

Specific hydrolysis rates, calculated for each reactor on the basis of COD balances and VS concentrations, were 0.063 ± 0.01 and 0.052 ± 0.01 g COD g^−1^VSd^−1^, respectively. These values were comparable to the one reported by Guo et al. [32], who operated a CSTR at higher HRT (12d) and lower OLR (4.4 g COD L^−1^d^−1^). The low values obtained in this study can be attributed to the high stabilisation degree of the WAS used.

Soluble-slime EPS were extracted from the effluents after 17 and 30 days for R-I, and 24 and 52 days for R-II, in order to follow the sEPS production dynamics (Table 2). The amount recovered in each extraction increased over time, while the COD to EPS ratio remained in the range of 1.31–1.45. Slight acidic conditions and sludge floc disintegration promoted soluble-slime EPS release into the supernatant during the operation. On the contrary, Guo et al. [32] experienced a stable and low level of the slime fraction of EPS during batch anaerobic digestion of WAS, due to its possible conversion into methane.

The sEPS in Reactor I at the end of the test (characterised by a sCOD value of 310 ± 25 mg L^−1^, half of the total sCOD in the reactor) was composed of 55% proteins and 40% carbohydrates, with a recovery of 4.7 ± 0.8 mg g^−1^ COD_fed_. In Reactor II, where the acidogenesis was more effective and stable, the sEPS was lower, namely 2.7 ± 0.5 mg g^−1^ COD_fed_, probably due to their transformation into VFAs. The high protein-to-carbohydrate ratio in the sEPS, which can likely be ascribed to the high sludge age of the activated sludge process [33], can provide high binding capacity to metals due to the larger molecular size of proteins with respect to polysaccharides [34].

In this study, the soluble concentration of As, Be, Cu, Ni, V, and Zn in the reactors during the tests was significantly correlated (*p* < 0.05) with the sEPS concentration extracted from the effluents, and the association was very strong (Pearson correlation coefficient between 0.80 and 0.93). Arsenic, with good mobility, tended to migrate to the supernatant [35] and can be easy to remove. It is interesting to note that in a previous study the authors found that the solubilisation of elements such as As, Ni, V, and Zn was strictly related to the degree of sludge disintegration with pre-treatments such as ultrasounds and thermal hydrolysis [27]. These metals, in fact, are progressively released into the soluble phase by increasing organic matter solubilisation and floc conformational change, confirming that the solubilisation of the EPS can lead to the release of adsorbed metals [36].

### 3.3. Microbial Community Analysis

**Figure 3:**
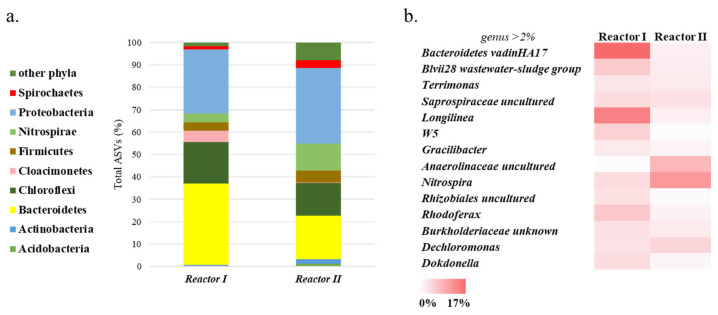
Bacterial microbiome estimated by high throughput 16 S rRNA gene sequencing in R-I and R-II sampled at the end of the operations at taxonomical phylum (**a**) and genera (**b**) levels. Only taxonomy groups having ≥2% of abundance in at least one sample are shown.

**Figure 4:**
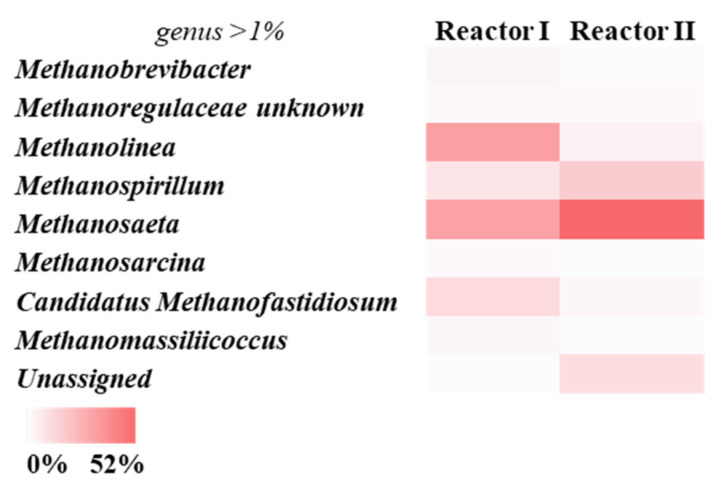
Frequency heat map of archaeal communities at taxonomical genera levels (only taxonomy groups having ≥1% of abundance in at least one sample are shown) observed in Reactor I and II. The colour intensity in each cell shows the relative ASV abundance.

Bacterial and archaeal 16S rRNA high throughput sequences analysis was performed on representative samples taken from both reactors at the end of the tests (Figure 3 and Figure 4). Diversity estimators—including the Simpson index (1-D), Shannon index (H) and Equitability index (J)—were used to investigate the diversity of the microbiome, and the main outputs are shown in Table 3.

The results showed that the bacterial biodiversity was higher in R-II than R-I, considering all the investigated indexes; by comparison, for the archaeal community, the higher biodiversity was obtained in R-I.

Bacterial analysis revealed that *Bacteroidetes*, *Chloroflexi*, *Proteobacteria*, and *Firmicutes* represented the core microbiome inhabiting both reactors (accounting for 73% and 87% of the total ASVs, for R-I and R-II, respectively, Figure 3a).

*Bacteroidetes* and *Proteobacteria* were the major phyla retrieved in both reactors (36% and 29% in R-I and 19% and 34% in R-II, respectively, Figure 3a) and have previously been described as key microorganisms for hydrolysis and VFA production from various substrates under anaerobic conditions [28]. The *Bacteroidetes* phylum was found in systems where hydrolysis and acidification of the substrate occurred [37]. Moreover, *Bacteroidetes* can potentially release more proteinaceous EPS—which aided its establishment in the reactor [38,39]—so their abundance in R-I can be correlated with the high amount of protein released into the effluent of this system (Table 2). These taxa included *Bacteroidetes vadinHA17* and *Rikenellaceae* families (which contains *Blvii28 wastewater-sludge group*; Figure 3b). The latter groups were composed of hydrolytic anaerobic bacteria [40], and their presence, in particular in R-I, can also be associated with the higher hydrolysis rate observed for this reactor (0.063 ± 0.01 g COD g^−1^VS d^−1^).

Conversely, the abundance of ASVs associated with the *Proteobacteria* phylum, in particular in R-II, was principally affiliated to aerobic or facultative anaerobic microorganisms (such as *Rhodoferax*, *Dechloromonas*, and *Dokdonella* genera, Figure 3b) that were reported as predominant microorganisms in activated sludge [41,42]. It is well known that the presence of these microorganisms in anaerobic reactors—in addition to members of the *Nitrospira* phylum (up to 12% in R-II)—can be due to the short HRT imposed on the systems. In fact, their appearance as major populations in these processes is likely due to incomplete digestion, unlike other populations, such as methanogens, syntrophs, and fermenters [42].

The *Chloroflexi* phylum represented the third most abundant anaerobic group found in the investigated systems (about 18% and 15% of the total ASVs in R-I and R-II, respectively. Figure 3a), followed by *Firmicutes* (4% and 5% of the total ASVs, Figure 3a). *Chloroflexi* and *Firmicutes* play a clear role in hydrolysis and acidogenesis, and their relevant presence may be due to short HRTs imposed [32]. In particular, *Chloroflexi*—reported to be responsible for carbohydrate degradation in anaerobic digesters [43]—was more abundant in Reactor I in which a more intense degradation occurred. The *Longilinea* genus represented about 15% of the total sequences affiliated to this phylum in R-I (Figure 3b). This genus is characterised by strictly anaerobic filamentous microorganisms and studies demonstrated that its growth is enhanced in co-cultivation with hydrogenotrophic methanogens [44]; therefore, its abundance can be attributed to the presence of this type of metabolism (Figure 4).

Members of *Firmicutes* phylum have been identified to hydrolyse and ferment large numbers of organic compounds under different anaerobic conditions [45,46]. In fact, they were more abundant in Reactor II, in which the production of VFAs was higher (acidification degree of 23 ± 2%, Figure 3a).

The relative abundance of *Cloacimonetes* (about 5% of the total ASVs) in Reactor I (only 0.07% of the sequences affiliated to this phylum was observed in R-II) suggests the possible presence of a syntrophic oxidation of the propionate pathway in this system [47]. This evidence could therefore explain the low acidification degree in R-I (only 9%): the presence of these microorganisms may prevent the accumulation of VFA (in particular propionate) due to the synergistic work with the hydrogenotrophic methanogens also present in the system.

Regarding the archaeal profile, almost all the found sequences were affiliated with the phylum *Euryarchaeota* for both reactors (about 95% and 80% of the total ASVs, respectively. Figure 4). As previously mentioned, genus level microbial community analysis revealed that acetoclastic and hydrogenotrophic metabolism coexisted in R-I. In fact, *Methanosaeta* were observed for 32% of the total ASVs, whereas *Methanolinea*, *Methanospirillum*, and *Candidatus Methanofastidiosum*—microorganisms associated with the production of methane from hydrogen—were present for 33%, 9%, and 12% of the total ASVs, respectively (Figure 4). By comparison, *Methanosaeta*, a well-known taxon capable of performing acetate degradation to produce CH_4_, was predominant in R-II, covering 52% of the total ASVs. Their significant presence in R-II was likely a result of increased concentration of acetic acid, indicating that acetoclastic methanogenesis was significant in this reactor [48].

The higher archaeal biodiversity (Table 3), together with higher methane yield of R-I with respect to R-II, may be correlated with the possibility of producing methane from different substrates. Moreover, the structural shift from hydrogenotrophic to acetoclastic methanogens observed between R-I and R-II was probably due to the selective pressures imposed on the microbial population by the reduction in HRT in the second system investigated [32].

## 4. Conclusions

The results of this study shed light on the EPS release from a low-cost feedstock, such as waste activated sludge, during the anaerobic fermentation stage carried out at high OLR and short HRT. The organically bound toxic metals, such as As, Be, Cu, Ni, V, and Zn, were released due to the disruption of the EPS matrix, thereby increasing the overall metal removal efficiency due to the action of hydrolytic microorganisms in a “cascade biorefinery” perspective. In fact, the properties of the EPS (biodegradable, rich in proteins and carbohydrates, easily extractable) make them attractive biopolymers that can be used as bioflocculants and cost-effective adsorbents, with a significant impact in transforming WWTPs into resource recovery facilities. However, there is still a need to identify the best operating conditions (HRT, OLR, type of waste activated sludge) to maximise the extraction of EPS and minimize the production of methane while maintaining the effectiveness of metal extraction.

## Figures and Tables

**Table 1:** Average characteristics of the feedstocks used for R-I and R-II.

	Feed R-I	Feed R-II
TS (g L^−1^)	43.6 ± 1	33.4 ± 7.1
VS (g L^−1^)	27.1 ± 1.1	23.6 ± 4.5
VS/TS (%)	62 ± 1	71 ± 3
COD_t_ (g L^−1^)	44.7 ± 5	38 ± 8
COD_1.2µm_ (mg L^−1^)	325 ± 31	205 ± 68
sCOD (mg L^−1^)	275 ± 28	80 ± 25
Sol Proteins (mg COD L^−1^)	203 ± 15	78 ± 18
Sol Carbohydrates (mg COD L^−1^)	24 ± 2	8.5 ± 5
N-NH_4_ (mg L^−1^)	90 ± 10	105 ± 10
P tot (g kg^−1^ TS)	18.6 ± 1.8	15.8 ± 1.5
N tot (g kg^−1^ TS)	50 ± 3	51 ± 2
BMP (mL CH_4_ g^−1^ VS_fed_)	0.11 ± 0.01	

**Table 2:** Characteristics of effluent produced in Reactor I and Reactor II, in two different sampling times.

	Reactor I		Reactor II	
Effluent	Day 17	Day 30	Day 24	Day 52
COD_1.2μm_ (mg L^−1^)	1450	1100	410	598
Sol Proteins (mg COD L^−1^)	756	1040	270	336
Sol Carbohydrates (mg COD L^−1^)	55	86	33	39
Soluble COD (mg L^−1^)	1300	650	250	410
VFA (mg COD L^−1^)	550	52	10	95
S-EPS (mg L^−1^)	75	220	38	110
Yield (mg sEPS g^−1^ COD_fed_)	1.5	4.7	1.1	2.7
Soluble P (mg L^−1^)	120	137	88	130
Total COD (g L^−1^)	29 ± 1.5	30 ± 2	29.5 ± 1.5	27.5 ± 1.5

**Table 3:** Statistics analysis of the microbial 16 S rRNA gene libraries obtained from the high-throughput 16 S rRNA gene sequencing.

		*Simpson_1-D*	*Shannon_H*	*Equitability_J*
*Bacteria*	R-I	0.9274	3.17	0.7743
	R-II	0.9735	4.56	0.8308
*Archaea*	R-I	0.7647	1.77	0.6535
	R-II	0.6807	1.66	0.5108

## Data Availability

Not applicable.
